# E-Learning challenges and trainee teacher engagement: Lessons from the COVID-19 pandemic

**DOI:** 10.1192/j.eurpsy.2024.1135

**Published:** 2024-08-27

**Authors:** Z. Boumaaize, A. Bouhaba, H. Guider, M. A. Lafraxo, Y. El Madhi, H. Hami

**Affiliations:** ^1^Laboratory of Biology and Health, Faculty of Sciences, Ibn Tofail University, Kenitra; ^2^Regional Center for Education and Training Professions, Rabat, Morocco

## Abstract

**Introduction:**

The sudden emergence of the COVID-19 pandemic resulted in a compulsory shift to distance learning due to school closures. Consequently, all educational courses were delivered virtually. To facilitate this transition for students and educators, the Ministry of Education implemented digital learning platforms, such as Taalim.ma and TelmidTice. Furthermore, the Broadcasting and Television National Company reorganized its television channels to maintain continuity throughout the academic year. As a result, trainee teachers have effectively shifted to distance learning, using resources such as e-Takwine, MOOC classes, and digital classrooms.

**Objectives:**

This study examines the factors that cause discomfort among trainee teachers and evaluates their influence on satisfaction with distance learning during the COVID-19 pandemic.

**Methods:**

We conducted a descriptive study with 370 Moroccan trainee teachers, with a mean age of 28.30 ± 5.99 years. We collected data using a self-administered questionnaire that was divided into three sections. The initial section examined sociodemographic and professional factors, including gender, age, marital status, academic level, and training cycle. The subsequent section focuses on discomfort-inducing factors, including living arrangements during lockdown, digital skills, material availability, network connectivity, and training schedules. Finally, the concluding section analyzes the trainees’ satisfaction and perceptions within their learning community.

**Results:**

The study indicated that 75% of trainee teachers lived in homes with four to seven residents, leading to confined living arrangements. Furthermore, 55% of the participants did not have a designated workspace for studying, focusing, or engaging with instructors. Participants identified various obstacles to online learning, including 80% lacking digital skills, 60% having insufficient equipment, 73.33% experiencing connection and network issues, 78.33% being unfamiliar with online learning, and 68.33% facing an unsuitable training schedule. The study indicates a positive correlation between trainee satisfaction and engagement (r = 0.422, p< 0.001).

**Conclusions:**

The COVID-19 pandemic has necessitated online education as a crucial resource for ensuring the continuity of education. This study highlights the paramount significance of developing effective e-training policies to direct novice teachers toward successful online learning by alleviating their discomfort factors.

**Disclosure of Interest:**

None Declared

